# Tezepelumab in severe asthma: chest computed tomography assessment of airway remodeling and clinical remission

**DOI:** 10.3389/fphar.2026.1757754

**Published:** 2026-01-30

**Authors:** Chiara Lupia, Caterina Battaglia, Daniela Pastore, Youngjin Lee, Giovanna Lucia Piazzetta, Emanuela Chiarella, Nadia Lobello, Giovanni Sireno, Alessandro Pullano, Claudia Crimi, Alessandro Vatrella, Francesco Manti, Pier Paolo Arcuri, Domenico Laganà, Girolamo Pelaia, Corrado Pelaia

**Affiliations:** 1 Department of Health Sciences, University “Magna Graecia” of Catanzaro, Catanzaro, Italy; 2 Department of Experimental and Clinical Medicine, University “Magna Graecia” of Catanzaro, Catanzaro, Italy; 3 Department of Radiological Science, Gachon University, Incheon, Republic of Korea; 4 Department of Medical and Surgical Sciences, University “Magna Graecia” of Catanzaro, Catanzaro, Italy; 5 Department of Clinical and Experimental Medicine, University of Catania, Catania, Italy; 6 Department of Medicine, Surgery and Dentistry, University of Salerno, Salerno, Italy; 7 Radiology Unit, “Renato Dulbecco” University Hospital, Catanzaro, Italy

**Keywords:** chest computed tomography, clinical remission, mucus plug, severe asthma, tezepelumab

## Abstract

**Introduction:**

Severe asthma is characterized by persistent uncontrolled symptoms despite optimal standard therapy and/or by the need for high-intensity treatment to maintain control. Thymic stromal lymphopoietin (TSLP) is a key innate cytokine that promotes bronchial inflammation in both T2-high and T2-low asthma, thus representing a suitable therapeutic target. Tezepelumab, a human monoclonal antibody designed to block TSLP, has been shown to be able to dampen airway inflammation and improve patients’ symptoms and overall outcomes.

**Methods:**

This study evaluated the effectiveness of tezepelumab in patients with severe asthma in a real-world clinical setting, examining its capacity to induce clinical remission and improve radiological markers of disease. Twenty patients with severe uncontrolled asthma received subcutaneous tezepelumab at a dose of 210 mg every 4 weeks. Clinical, functional, biologic, and radiological data were collected at baseline and after 12 months of treatment.

**Results:**

The results showed a significant increase in Asthma Control Test (ACT) score, from baseline 11.70 ± 1.78 to 20.15 ± 1.38 (p < 0.0001). Oral corticosteroid (OCS) intake decreased from 12.50 mg/day (5.00–25.00) to 0.00 mg/day (0.00–3.75) (p < 0.0001), and the rate of yearly exacerbations lowered from 2.0 (2.0–3.0) to 0.0 (0.0–1.0) (p < 0.0001). There was also a notable increase in pre-bronchodilator forced expiratory volume in 1 s (FEV_1_), from baseline 57.15% ± 17.18% to 66.35% ± 18.09% (p < 0.001). Clinical remission was achieved by 30% of patients, while 55% reached partial remission. Radiologically, the Mucus Plug Score (MPS) decreased from 5.36 ± 2.85 to 3.94 ± 2.69 (p < 0.0001), and radiological metrics showed inverse correlations with functional parameters. In simple logistic regression analysis, lower MPS values at baseline and at follow-up were significantly associated with higher odds of achieving complete clinical remission at 12 months of add-on tezepelumab therapy.

**Conclusion:**

In summary, tezepelumab induced significant benefits in patients with severe asthma in this real-world cohort, improving symptoms, lung function, and radiological outcomes.

## Introduction

Asthma is a chronic inflammatory disease of the respiratory tract, characterized by reversible airflow obstruction and a clinical history of dyspnea, wheezing, chest tightness, and cough (Global Strategy, 2025). Severe asthma is a disease variant that remains uncontrolled despite maximally optimized standard therapy and/or requires high-intensity treatment to prevent it from becoming uncontrolled. Approximately 300 million individuals worldwide are affected by asthma, with severe disease accounting for about 5%–10% of cases (Côté et al., 2020). Asthma is a multifactorial airway disorder influenced by both individual predisposition (e.g., atopy, genetic background) and environmental factors (Porsbjerg et al., 2023). Bronchial epithelial cells release alarmins such as thymic stromal lymphopoietin (TSLP), IL-25, and IL-33, which activate ILC2s and amplify type 2 inflammation (Moore et al., 2007; Pastore et al., 2025). This endotypic profile is typically associated with early onset, atopy, peripheral blood eosinophilia, and corticosteroid responsiveness, and represents the main target of currently available biologic therapies (Lambrecht and Hammad, 2015). Despite the wide range of therapeutic options currently available for T2-high asthma, effective treatments for T2-low asthma remain limited. In this context, TSLP plays a central role (Soumelis et al., 2002). TSLP is an epithelial-derived cytokine released in response to environmental stimuli, including allergens, cigarette smoke, and viral or bacterial infections. It exists as two isoforms: a constitutively expressed short form (60 amino acids) and a long variant (159 amino acids) overexpressed under inflammatory conditions, which is markedly elevated in asthma. TSLP contributes to Th2 differentiation, mast cell and basophil degranulation, eosinophil survival and migration, ILC2 activation, and airway remodeling through stimulation of fibroblasts and smooth muscle cells. Circulating TSLP levels correlate with airflow limitation and clinical severity, highlighting the potential of this alarmin as both a biomarker and a therapeutic target in severe asthma (Gauvreau et al., 2020). The primary objectives of asthma management are symptom control and prevention of exacerbations, typically achieved through inhaled corticosteroids (ICS) alone or in combination with long-acting β_2_-agonists (LABA) (Chung et al., 2014). In adults with severe asthma, the addition of a long-acting muscarinic antagonist (LAMA), such as glycopyrronium in fixed triple combinations (e.g., beclomethasone–formoterol–glycopyrronium), may further improve lung function (Maiorano et al., 2025; Maiorano et al., 2024). When conventional therapy is insufficient, biologic agents targeting key inflammatory pathways are considered (Porsbjerg et al., 2018). Among these, tezepelumab—a fully human monoclonal antibody directed against TSLP—prevents its interaction with the TSLP receptor complex and has demonstrated efficacy in both T2-high and T2-low asthma (D'Amato et al., 2025). By inhibiting TSLP, tezepelumab suppresses ILC2 production of IL-5 and IL-13, Th2 cell polarization, eosinophil survival, and mast cell/basophil activation. It also modulates Th17-driven neutrophilic inflammation, suggesting broad efficacy across asthma endotypes (Corren et al., 2017).

The concept of clinical remission in asthma remains under active debate. According to the Severe Asthma Network Italy (SANI), complete clinical remission is defined as a stable disease state lasting at least 12 months, characterized by full symptom control, with an Asthma Control Test (ACT) score ≥20 or an Asthma Control Questionnaire (ACQ) score <1, absence of systemic corticosteroid use, no exacerbations, and stable or normalized lung function, with a forced expiratory volume in one second (FEV_1_) ≥80% predicted. On the other hand, partial clinical remission includes the first two criteria and at least one of the latter two (Canonica et al., 2023; Ca et al., 2024). Severe asthma is associated not only with various comorbid conditions, but also with relevant radiological abnormalities. Radiological features of this disease encompass bronchial wall remodeling, bronchiectasis, and mucus plugs (Thomas et al., 2022). Mucus plugging, resulting from changes in mucus composition and ineffective clearance, impairs airflow and causes shifts in clinical symptoms (Kim et al., 2018; Park et al., 1997). In severe asthma cases, these mechanisms contribute to mucus blockage, which may lead to symptoms such as wheezing and shortness of breath (Jensen et al., 2002; Dunican et al., 2018a). Ciliary dysfunction and dysbiosis are major contributors to the disease, while mucus plugs exacerbate asthma by worsening airway obstruction and facilitating bacterial infections (Huang et al., 2015). Chest high-resolution computed tomography (HRCT) enables non-invasive identification and quantification of mucus plugs, and the Mucus Plug Score (MPS) developed by Dunican et al. correlates with airway limitation and disease severity (Dunican et al., 2018b). Although imaging-based indicators have allowed for a more precise assessment of structural modifications in severe asthma, these pathological changes are often not sufficiently improved by conventional therapies alone.

Our present study aimed to assess in patients with severe asthma the real-world effectiveness of tezepelumab, with regard to the achievement of both clinical remission and radiological improvement after 1 year of treatment.

## Materials and methods

### Study population

Twenty patients with severe asthma were recruited at the outpatient Units of Pulmonology and Radiology, “Magna Graecia” University Hospital of Catanzaro, Italy. Each patient had a confirmed diagnosis of asthma, associated with appropriately identified and managed comorbidities. Inhalation techniques and treatment adherence were verified as well as pharmacological management was optimized. All participants reported persistent asthma symptoms and required high doses of ICS/LABA/LAMA combinations, in addition to an almost continuous oral corticosteroid (OCS) regimen. Maintenance inhaled corticosteroid therapy consisted of beclomethasone dipropionate 172 μg per inhalation, administered as two inhalations twice daily, and the regimen remained unchanged throughout follow-up. Enrolment criteria included the following features: diagnosis of severe uncontrolled asthma, according to American Thoracic Society (ATS) and European Respiratory Society (ERS) guidelines (Chung et al., 2014); at least two exacerbations in the previous year treated with systemic corticosteroids or requiring hospitalization, despite maximal inhaled therapy (GINA step 4–5 treatment), or, in addition to inhaled therapy, a continuous OCS use for at least 6 months; age over 18 years; availability of HRCT scans performed before and 1 year after starting tezepelumab therapy; good-quality CT images. Tezepelumab was administered subcutaneously at a dose of 210 mg every 4 weeks. This observational study adhered to both Good Clinical Practice (GCP) standards and principles of the Declaration of Helsinki, and was approved by the Ethics Committee of Calabria Region (Catanzaro, Italy; authorization no. 9, 11 January 2024).

### Data collection and evaluation

Clinical, functional, biological, and radiological data were collected at both baseline and after 1 year of add-on anti-TSLP treatment. All patients underwent a comprehensive assessment covering their medical history and a physical examination. The focus was addressed on the age at which asthma first appeared, smoking status (never, current, or former), history of acetylsalicylic acid intolerance or adverse drug reactions, and body mass index (BMI), calculated by dividing weight by height squared. Chronic rhinosinusitis (CRS), with or without nasal polyps (CRSwNP and CRSsNP, respectively), and gastroesophageal reflux disease (GERD) were identified as comorbid conditions. The frequency of clinically significant exacerbations in the previous year was recorded, defining these events as episodes of asthma worsening requiring systemic corticosteroids for more than 3 days and/or hospitalization. A standard panel of inhalant allergens was used for skin prick testing, and the ImmunoCAP system (Phadia, Uppsala, Sweden) was utilized to measure total serum IgE levels. An automated hematology analyzer was used to determine the eosinophil count in peripheral blood. ACT is a 5-item questionnaire designed to assess asthma control. A Likert scale from 1 to 5 was used to evaluate each item, with 1 indicating the lowest score and 5 the highest one. Patients were stratified according to inflammatory endotype. Any of the following criteria were used to determine the eventual presence of underlying T2 inflammation (T2-high): (i) at least two fractional exhaled nitric oxide (FeNO) assessments with values of 25 ppb or above; (ii) at least two blood eosinophil measurements with counts of 150 cells/μL or higher; and (iii) clinically significant allergies with related sensitization.

According to ATS/ERS guidelines (Chung et al., 2014), spirometry was performed both before and after administration of 400 μg of salbutamol as a fast-acting bronchodilator. We considered the following pulmonary function parameters: FEV_1_, forced vital capacity (FVC), total airway resistance (R_tot_), residual volume (RV), forced expiratory flow between 25% and 75% of FVC (FEF_25–75_), and diffusion lung capacity for carbon monoxide (DLCO). The best of three spirometric attempts was recorded, and the results were presented as both absolute values and percentages of predicted measures. Spirometry and body plethysmography were performed using the MasterScreen and MasterScreen Body systems (Jaeger-Viasys; CareFusion, Höchberg, Germany), fully complying with ATS/ERS guidelines (Chung et al., 2014).

### Calculation of mucus plug score and airway analysis

MPS was determined by systematically evaluating each segment of every pulmonary lobe, in order to identify the presence or absence of mucus plugs. Each segment was assigned a score of 1 when a mucus plug was detected and 0 when it was absent. The total score, obtained by summing all segmental values, provided a global measurement for both lungs, ranging from 0 to 20. To minimize potential errors associated with the small size of distal airways, segments located within 2 cm of the diaphragmatic and costal pleura were excluded from the analysis, as accurate assessment of mucus occlusion in these regions is challenging.

**FIGURE 1:**
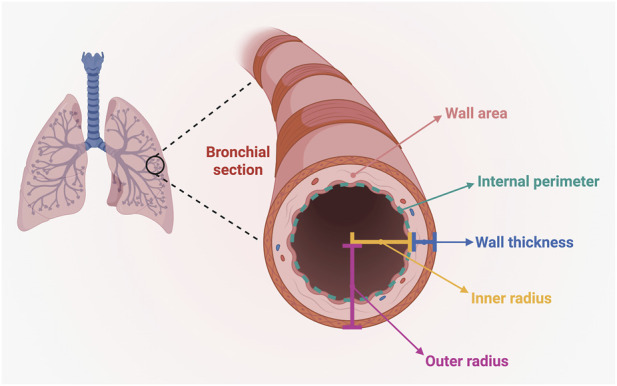
Radiological parameters evaluated on bronchial section. This original figure was created by the authors using “BioRender.com”.

The images were obtained with CT scan (multi-slice Toshiba Aquilion 64, Otawara, Japan), using 1-mm axial slices with specific iterative reconstruction algorithm. These evaluations were performed using 3D Slicer, version 5.2.2 (3D Slicer, Boston, MA, USA), which is a free and open-source software platform for medical image visualization and processing developed at Brigham and Women’s Hospital (Harvard Medical School, Boston, MA, USA) in collaboration with international institutions. Images were imported in DICOM format, and lung segmentation was carried out using the *Lung CT Segmentation* extension. Subsequently, the *Airway Inspector* tool was applied to the segmented airways, with the aim of obtaining several quantitative measurements and parameters related to airway morphology and function. In particular, the measured parameters included inner radius, outer radius, wall thickness, wall area, internal perimeter, luminal area, cross-sectional area, and wall intensity. This last parameter was defined as the mean CT attenuation (HU) within the airway wall compartment (region between inner and outer contours) extracted by *Airway Inspector*. Segmentation was performed automatically and then visually inspected by 2 readers (radiologists). Airway morphometry was quantified on inspiratory CT at standardized central airway levels. Measurements were obtained in segmental (third-generation) and subsegmental (fourth–fifth-generation) bronchi. The most significant radiological metrics measured on bronchial sections are presented in Figure 1.

### Statistical analysis

Data normality was assessed by performing the Anderson-Darling and Kolmogorov-Smirnov tests. Mean ± standard deviation (SD) was used to represent normally distributed data, whereas median values with interquartile range (IQR) were applied to report skewed data distributions. Data were expressed as mean ± standard deviation (SD) when normally distributed, otherwise as median values with interquartile range (IQR). The choice of tests was based on data distribution: parametric or non-parametric. When applicable, the Wilcoxon signed-rank test and paired t-test were used to compare variables. The overall study population was stratified according to T2 biomarker levels (T2-high/T2-low). A p-value <0.05 was considered as statistically significant. Associations between radiological and functional parameters were evaluated using Spearman’s rank correlation test, and correlation coefficients (r) were calculated. Simple logistic regression analyses were performed to evaluate the association between MPS values and the likelihood of achieving complete clinical remission after 12 months of add-on tezepelumab therapy. Statistical analyses and figures were generated using Prism software, version 10.3.0 (GraphPad Software Inc., San Diego, CA, USA).

## Results

Twenty participants were enrolled, including 12 women (60%) and 8 men (40%), with a mean age of 63.75 ± 13.25 years and a mean body mass index (BMI) of 26.93 ± 4.64 kg/m^2^. Among them, 10 patients (50%) exhibited a T2-high endotype. The mean baseline FEV_1_ was 57.15% ± 17.18% of the predicted normal value, while the residual volume (RV) was 113.80% ± 48.55%. The mean MPS value was 5.36 ± 2.85. Baseline patient characteristics are summarized in Table 1. All the included patients were naïve with respect to previous biological therapies for severe asthma.

**TABLE 1:** Baseline patient characteristics.

Age, mean ± SD, years	63.75 ± 13.25
Male sex, N (%)	8 (40)
Weight, mean ± SD, kg	72.89 ± 13.17
Height, mean ± SD, cm	163.16 ± 10.09
BMI, mean ± SD, kg/m^2^	26.93 ± 4.64
Smokers and ex-smokers, N (%)	4 (20)
FEV1/FVC, mean ± SD, ratio	58.66 ± 16.26
CRSwNP, N (%)	1 (5)
GERD, N (%)	9 (45)
T2-high, N (%)	10 (50)
Atopy, N (%)	4 (20)
Post-bronchodilator reversibility, N (%)	12 (60)

Abbreviations: BMI, body mass index; forced expiratory volume in 1 s, FEV_1_; forced vital capacity, FVC; CRSwNP, chronic rhinosinusitis with nasal polyps; GERD, gastroesophageal reflux disease; SD, standard deviation.

**FIGURE 2:**
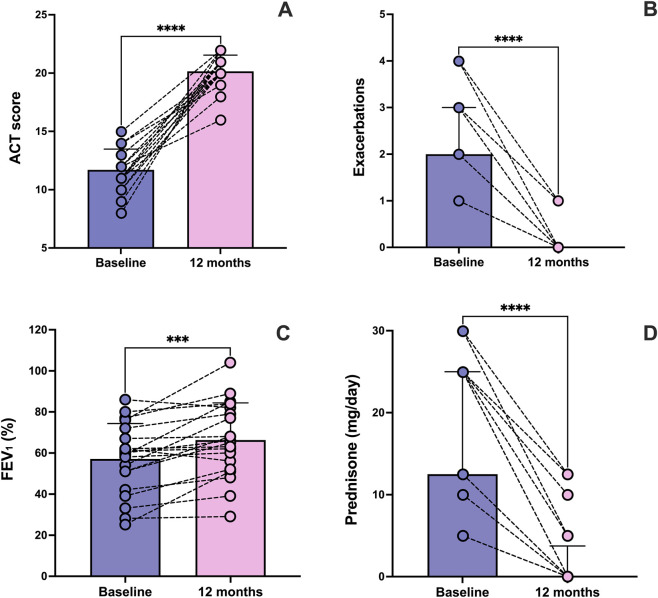
Twelve-month effects of tezepelumab on asthma control test (ACT) score **(A)** exacerbation rate **(B)** forced expiratory volume in 1 s (FEV_1_) **(C)** and oral corticosteroid (OCS) use **(D)**. Values of ACT score and FEV_1_ are expressed as mean (±SD). Values of exacerbation rate and OCS use are expressed as median (IQR). ***p < 0.001; ****p < 0.0001.

**FIGURE 3:**
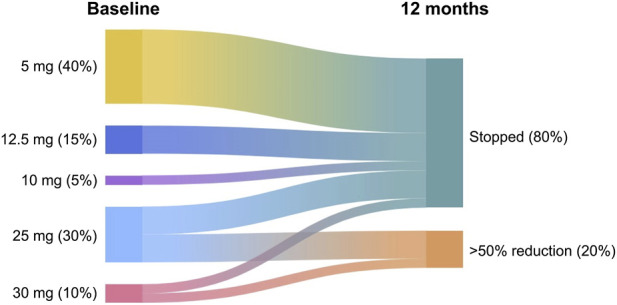
Sankey plot illustrating changes in oral corticosteroid (OCS) intake from baseline to 12 months after initiation of tezepelumab.

With regard to ACT score, a significant improvement was observed, shown by the increase from a baseline value of 11.70 ± 1.78 to 20.15 ± 1.38 after 1 year of treatment (p < 0.0001) (Figure 2A). Compared with the 12 months preceding the first injection of tezepelumab, the median number of asthma exacerbations markedly decreased from 2.0 (2.0–3.0) to 0.0 (0.0–1.0) after 1 year of anti-TSLP therapy (p < 0.0001) (Figure 2B). These clinical effects of tezepelumab were paralleled by a clear improvement in pulmonary function. Indeed, after 1 year of add-on biological therapy, pre-bronchodilator FEV_1_ significantly increased from 57.15% ± 17.18% at baseline to 66.35% ± 18.09% (p < 0.001) (Figure 2C). During the same period, this substantial improvement in asthma control allowed for a progressive reduction of OCS intake; over 1 year, the median daily prednisone use decreased from a baseline dose of 12.50 mg/day (5.00–25.00) to 0.00 mg/day (0.00–3.75) (p < 0.0001) (Figure 2D). At baseline, 20 patients (100%) were using OCS. Figure 3 displays the OCS doses (in prednisone equivalent) and their changes at follow-up. By the follow-up, 80% of patients had stopped using OCS, and an additional 20% experienced a reduction of over 50% in their dose. Based on these findings, complete clinical remission after 1 year of tezepelumab treatment was achieved in 6 patients (30%), while 11 patients (55%) achieved partial remission. Among the 17 subjects who achieved complete or partial clinical remission, 9 (53%) had a T2-high asthma endotype, whereas 8 (47%) had a T2-low endotype.

Moreover, R_tot_ significantly decreased from 0.55 ± 0.32 kPa·s/L at baseline to 0.46 ± 0.17 kPa·s/L (p < 0.05). Over the same period, FEF_25–75_ increased from a baseline value of 1.05 ± 0.62 L/s to 1.30 ± 0.83 L/s (p < 0.05). FVC also improved significantly, rising from 2.20 ± 0.84 L to 2.44 ± 0.91 L (p < 0.01). FVC increase was associated with a concomitant improvement of the diffusing capacity for carbon monoxide (DLCO), which rose from 5.27 ± 2.31 mmol/(min·kPa) to 5.70 ± 2.19 mmol/(min·kPa) (p < 0.05). Functional results are summarized in Table 2.

**TABLE 2:** Airway functional parameters before and after 1 year of treatment with tezepelumab.

Airway functional parameter	Baseline	Follow-up	p-value
FEV_1_, mean value ±SD, %	57.15 ± 17.18	66.35 ± 18.09	<0.001
FEV_1_, mean value ±SD, L	1.50 ± 0.58	1.72 ± 0.64	<0.001
FVC, mean value ±SD, %	65.05 ± 18.07	72.20 ± 18.73	<0.01
FVC, mean value ±SD, L	2.20 ± 0.84	2.44 ± 0.91	<0.01
FEV1/FVC, mean ± SD, ratio	58.66 ± 16.26	63.93 ± 17.31	<0.0001
R_tot,_ mean value ±SD, %	185.30 ± 107.60	153.10 ± 58.04	<0.05
R_tot,_ mean value ±SD, kPa·s/L	0.55 ± 0.32	0.46 ± 0.17	<0.05
RV, mean value ±SD, %	113.80 ± 48.55	100.10 ± 55.04	0.086
RV, mean value ±SD, L	2.37 ± 1.18	2.12 ± 1.32	0.134
FEF_25-75_, mean value ±SD, %	45.95 ± 25.40	57.15 ± 28.13	<0.05
FEF_25-75_, mean value ±SD, L/s	1.05 ± 0.62	1.30 ± 0.83	<0.05
DLCO, mean value ±SD, %	67.56 ± 22.00	73.22 ± 21.68	<0.01
DLCO, mean value ±SD, mmol/(min·kPa)	5.27 ± 2.31	5.70 ± 2.19	<0.05

Abbreviations: FEV_1_, forced expiratory volume in one second; FVC, forced vital capacity; R_tot_, total airway resistance; RV, residual volume; FEF_25-75_, forced expiratory flow between 25% and 75% of FVC; DLCO, diffusion lung capacity for carbon monoxide; SD, standard deviation.

**FIGURE 4:**
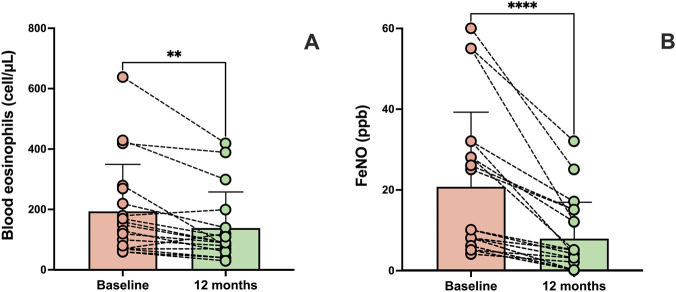
Effects of 12 months of tezepelumab treatment on blood eosinophil counts **(A)** and fractional exhaled nitric oxide (FeNO) levels **(B)**. Values of blood eosinophils and FeNO are expressed as mean (±SD). **p < 0.01; ****p < 0.0001.

After 1 year of anti-TSLP therapy, blood eosinophil count significantly decreased from 195.90 ± 161.60 cells/µL at baseline to 138.20 ± 119.80 cells/µL (p < 0.01) (Figure 4A), and FeNO levels dropped from 20.80 ± 18.45 to 7.95 ± 9.00 (p < 0.0001) (Figure 4B). Conversely, no statistically significant change was observed in total serum IgE levels, which declined from 44.00 IU/mL (21.00–78.40) at baseline to 28.50 IU/mL (10.00–65.00) after 1 year (p = 0.108).

**FIGURE 5:**
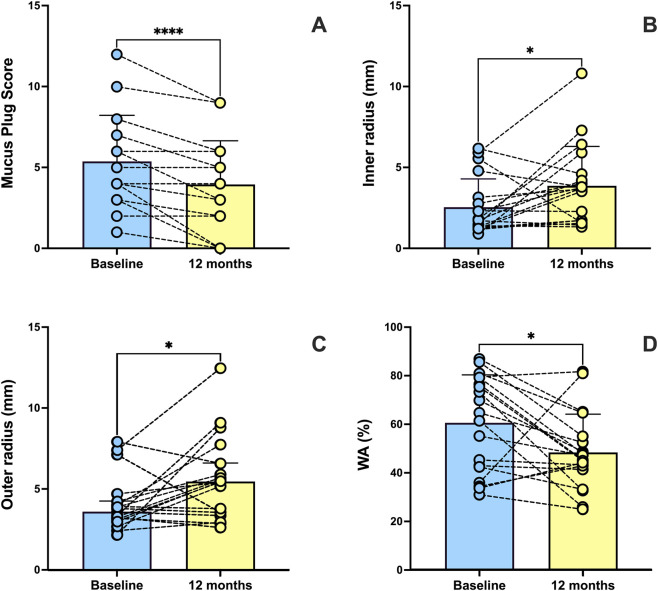
Effects of 12 months of tezepelumab treatment on Mucus Plug Score **(A)** inner bronchial radius **(B)** outer bronchial radius **(C)** and airway wall area (WA) percentage **(D)**. Values of Mucus Plug Score, inner radius and WA percentage are expressed as mean (±SD). Values of outer radius are expressed as median (IQR). *p < 0.05; ****p < 0.0001.

**FIGURE 6:**
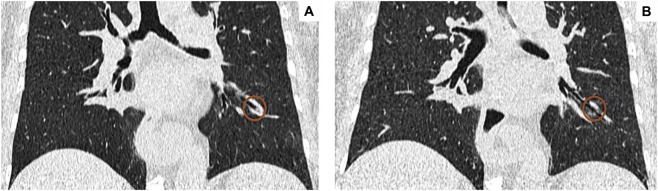
Effects of tezepelumab on mucus plugs as assessed by chest high-resolution computed tomography (HRCT) before **(A)** and after **(B)** 12 months of treatment.

From a radiological standpoint, a statistically significant MPS reduction was observed 12 months after starting treatment with tezepelumab. MPS decreased from 5.36 ± 2.85 at baseline to 3.94 ± 2.69 at one-year follow-up, thus undergoing a highly significant change (p < 0.0001) (Figure 5A). Through airway analysis performed using the “Airway Inspector” tool integrated in 3D Slicer, several parameters specifically related to airway anatomical and morphological characteristics were assessed. Table 3 shows the airway radiological parameters, recorded before and after 1 year of treatment with tezepelumab. Specifically, the inner bronchial radius increased from 2.54 ± 1.74 mm to 3.86 ± 2.43 mm (p < 0.05), while the outer radius incremented from 3.60 mm (3.14–4.25) to 5.46 mm (3.57–6.60) (p < 0.05) (Figures 5B,C). Moreover, a significant reduction was observed in the bronchial wall area percentage (WA%), which decreased from 60.61% ± 19.71% to 48.41% ± 15.76% after 12 months (p < 0.05) (Figure 5D). HRCT images illustrating the twelve-month effects of tezepelumab on mucus plugs are shown in Figure 6.

**TABLE 3:** Airway radiological parameters before and after 1 year of treatment with tezepelumab.

Airway radiological parameter	Baseline	Follow-up	p-value
Inner radius, mean value ±SD, mm	2.54 ± 1.74	3.86 ± 2.43	<0.05
Outer radius, median (IQR), mm	3.60 (3.14–4.25)	5.46 (3.57–6.60)	<0.05
Wall thickness, mean value ±SD, mm	1.69 ± 0.29	1.77 ± 0.39	0.554
Wall area, mean value ±SD, %	60.61 ± 19.71	48.41 ± 15.76	<0.05
Internal perimeter, mean value ±SD, mm	13.15 ± 7.49	16.12 ± 6.29	0.145
Luminal area, median (IQR), mm^2^	17.30 (7.40–41.60)	53.87 (15.60–61.00)	0.063
Cross-sectional area, median (IQR), mm^2^	49.12 (36.69–75.84)	94.57 (43.75–112.9)	0.060
Wall intensity, mean value ±SD, HU	−570.90 ± 200.8	−635.50 ± 201.7	0.378
Mucus plug score, mean value ±SD	5.36 ± 2.85	3.94 ± 2.69	<0.0001

Abbreviations: SD, standard deviation; IQR, interquartile range.

**FIGURE 7:**
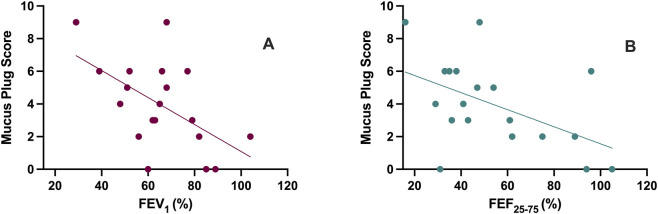
Correlation between Mucus Plug Score and forced expiratory volume in 1 s (FEV_1_) **(A)** and between Mucus Plug Score and forced expiratory flow between 25% and 75% of forced vital capacity (FEF_25–75_) **(B)** after 12 months of tezepelumab treatment.

**FIGURE 8:**
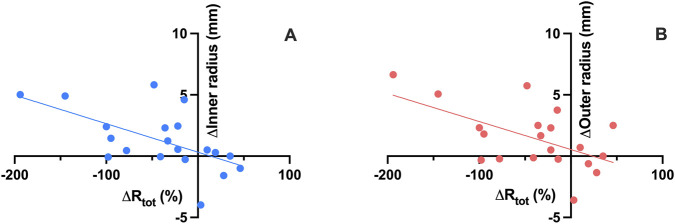
Correlation between total airway resistance (R_tot_) changes and inner radius variations **(A)** and outer radius variations **(B)** after 12 months of tezepelumab treatment.

After 12 months of tezepelumab therapy, a significant inverse correlation was observed between MPS and both FEV_1_ (%) (r = −0.554, p < 0.05) and FEF_25–75_ (%) (r = −0.502, p < 0.05) (Figures 7A,B). Furthermore, a significant inverse correlation was observed between R_tot_ changes and variations in inner radius (r = −0.591, p < 0.01), as well as outer radius (r = −0.566, p < 0.01) (Figures 8A,B). Simple logistic regression analysis showed that lower MPS values at baseline (OR = 0.46, p < 0.05) and at follow-up (OR = 0.43, p < 0.01) were significantly associated with a higher probability of achieving complete clinical remission after 12 months of add-on tezepelumab therapy.

Finally, we compared the principal clinical, functional, and radiological outcomes between the two distinct endotypes (T2-high and T2-low). After 12 months of tezepelumab therapy, improvements were comparable across the two patient subgroups. Specifically, mean changes in ACT score were 8.66 ± 1.73 points in T2-high patients and 8.63 ± 2.65 points in T2-low patients (p = 0.238). The daily prednisone dose decreased by −11.00 ± 5.46 mg in the T2-high group and by −13.86 ± 9.44 mg in the T2-low group (p = 0.134). The reduction in the annualized number of asthma exacerbations was −2.22 ± 0.83 in T2-high patients and −2.27 ± 0.90 in T2-low patients (p = 0.832). The increase in FEV_1_ was 8.66% ± 10.31% in the T2-high subgroup and 9.72% ± 10.70% in the T2-low subgroup (p = 0.934). Finally, the MPS decreased by −1.33 ± 1.22 points in T2-high patients and by −1.63 ± 1.28 points in T2-low subjects (p = 0.907).

## Discussion

The above results referring to our single-centre observational study, conducted in real-world clinical practice among patients with severe asthma, provide convincing evidence in further support of the therapeutic efficacy of tezepelumab. After 1 year of adjunctive treatment with this monoclonal antibody, significant clinical, functional, biologic and radiological ameliorations were observed, including clinical remission and a marked MPS reduction. Compared with baseline assessment, all patients exhibited a rapid and consistent improvement in asthma symptoms, as demonstrated by the significant increase in ACT score found 1 year after the first administration of tezepelumab. This questionnaire is often preferred in real-life settings due to its simplicity and ease of use. Our findings are consistent with those reported in recent studies carried out in real-world practice contexts (Biener et al., 2024; Carpagnano et al., 2025; Betancor et al., 2024). We detected a rapid and significant clinical improvement which allowed for an early and gradual reduction of the daily OCS dose. This real-world observation is particularly noteworthy. Lowering OCS dependence remains a key therapeutic aim in patients with severe asthma, who are often reliant on these drugs and therefore at risk of significant adverse effects, including osteoporosis, diabetes, hypertension, cataracts, and glaucoma (Heffler et al., 2019; Canonica et al., 2019). Regarding lung function, tezepelumab elicited a quick improvement in airflow limitation in the large airways, evidenced by a significant increase in pre-bronchodilator FEV_1_. The drug’s effectiveness in producing clinically meaningful FEV_1_ increments has been clearly documented in patients with severe eosinophilic asthma characterized by a T2-high phenotype. This benefit seems to be mediated by tezepelumab’s ability to substantially reduce airway eosinophilic infiltration in patients with moderate-to-severe uncontrolled asthma, as confirmed by the phase 2 CASCADE trial (Diver et al., 2021). Moreover, in our study significant FEV_1_ increases were also recorded in patients with severe asthma exhibiting a T2-low pattern. In both T2-high and T2-low subgroups, the improvement in airflow limitation was supported by a notable decrease in airway resistance compared with baseline values. This therapeutic efficacy is closely related to the upstream mechanism of action of tezepelumab, which inhibits the pleiotropic effects exerted by TSLP on many cellular populations, including innate lymphoid cells, T lymphocytes, dendritic cells, mast cells, basophils, eosinophils, and neutrophils (Dorey-Stein and Shenoy, 2021). Compared with the period before the first administration of tezepelumab, we observed a marked reduction in the median number of asthma exacerbations. This result is concordant with data referring to PATHWAY and NAVIGATOR clinical trials (Corren et al., 2017; Menzies-Gow et al., 2020). Taken together, these findings enabled us to identify a complete clinical remission in 30% of our patients, while partial remission was detected in 55%. Such observations are in agreement with several controlled trials and real-world studies, which confirm the achievement of clinical remission in patients with severe asthma treated with tezepelumab. A post-hoc analysis of both NAVIGATOR and DESTINATION trials demonstrated that patients with severe uncontrolled asthma treated with tezepelumab had a higher likelihood of experiencing a full clinical response and remission compared to those receiving placebo (Wechsler et al., 2024). In real-world research, Gates et al. found that tezepelumab led to significant clinical improvements and remission in up to 55% of patients with T2-high severe asthma (Gates et al., 2025). Furthermore, our results show that tezepelumab was effective in both T2-high and T2-low severe asthma phenotypes, thus corroborating the findings reported by two previous real-world studies (Poto et al., 2025; Pelaia et al., 2025).

We noticed that tezepelumab did not significantly affect total serum IgE concentrations, although it lowered both blood eosinophil counts and FeNO levels, consistently with the results of the NAVIGATOR study (Menzies-Gow et al., 2020). Unlike the CASCADE study, our real-world investigation documented a rapid beneficial effect of tezepelumab on small airways, as indicated by the notable increase in FEF_25–75_ (Diver et al., 2021). One of the key factors that may explain the early clinical and functional improvements observed in the present real-world setting is the positive impact of tezepelumab on airway obstruction due to mucus plugging. This relevant therapeutic effect appears to be driven by tezepelumab-induced upstream suppression of several pro-inflammatory pathways responsible for mucus overproduction (Chan and Lipworth, 2022; Mummy et al., 2022).

The second part of our real-world research specifically focused on MPS evaluation. In this regard, the study conducted by Dunican et al., in 2018 marked a turning point in diagnosing and quantifying mucus plugs using HRCT (Dunican et al., 2018a). MPS holds significant clinical importance because this score extends beyond a simple radiological assessment, and offers a quantitative measure of the pathophysiological contribution of mucus retention within the airways. Additionally, MPS provides complementary information to traditional FEV_1_-based assessment by capturing structural airway obstruction that functional measures often overlook, making it a promising tool for predicting therapeutic response and refining patient phenotyping in future research. Based on this methodology, we focused on MPS calculation (Dunican et al., 2018a). By summing the scores of individual lung segments, a total value ranging from 0 to 20 was obtained for each participant. To ensure high measurement reliability, peripheral airways of small calibre—where assessment of mucus occlusion is less precise—were excluded from evaluation. CT image analysis was performed using the 3D Slicer software, enabling advanced visualization and extraction of detailed parameters, which allowed comparison between pre-treatment and one-year post-treatment data. Our results agree with the post-hoc CT analysis of CASCADE clinical trial (Nordenmark et al., 2023), which first demonstrated the effectiveness of tezepelumab in reducing mucus plug load. After 28 weeks of therapy, participants receiving tezepelumab showed remarkable improvements compared to the placebo group. In our real-world cohort, tezepelumab elicited significant clinical benefits in terms of respiratory function, bronchial obstruction, and radiological outcomes. Following 12 months of treatment, we observed a significant inverse relationship between MPS and functional parameters, specifically FEV_1_ and FEF_25–75_. We also identified an inverse correlation between R_tot_ and radiological measures such as inner radius and outer radius. These findings suggest that increases in airway calibre are linked with reductions of total airway resistance, as well as with enhanced airflow in both central and small airways, thus aligning with the results of the aforementioned post hoc analysis (Nordenmark et al., 2023). Several prior studies have established a link between the use of biologic agents for severe asthma and improvements in radiological scores. For example, Honglei Shi et al. demonstrated that omalizumab reduced airway wall thickness and mucus plugging, thereby improving airflow limitation (Shi et al., 2024). Mozaffaripour further advanced this field by developing a new image-based index of pulmonary morphology, showing that in 5 out of 6 patients who achieved clinical remission, airway and vascular remodeling regressed after 2.5 years of anti-IL5Rα therapy (Mozaffaripour et al., 2025). Ongoing research is also further investigating the effects of tezepelumab. In his abstract, Norheim reported that anti-TSLP treatment reduced MUC5AC mucin expression independently of T2 biomarker status, through downregulation of EGFR pathway (Norheim et al., 2024). Moreover, our findings suggest that MPS may act as an early marker of treatment responsiveness, with lower scores identifying patients who are more likely to achieve deep disease control under tezepelumab. This observation supports the potential role of MPS in refining patient selection and monitoring severe asthma management in real-world.

Taken together, our findings offer real-world evidence supporting the clinical, functional and radiological significance of TSLP inhibition in severe asthma treatment.

## Conclusion

In conclusion, our observational study confirms and broadens, within the context of real-world respiratory clinical practice, the evidence previously emerging from randomized controlled trials regarding the effectiveness of tezepelumab in treating severe asthma. This biologic agent exerted a markedly positive therapeutic impact by reducing exacerbation frequency, decreasing OCS use, enhancing symptom control and improving lung function, thereby leading to clinical remission in a significant proportion of patients after 1 year of treatment. Additionally, our research supports and expands on previous evidence demonstrating improvements in radiological parameters in patients treated with tezepelumab.

MPS may represent an additional, mechanism-relevant imaging marker aimed to contextualize and potentially predict the therapeutic response to tezepelumab. Unlike blood eosinophils and FeNO, which primarily reflect systemic and airway type-2 inflammatory activity, the mucus plug score captures a structural–functional component of disease (airway lumen occlusion and impaired ventilation) that may persist despite fluctuations in conventional inflammatory biomarkers. However, we do not interpret our data as evidence that mucus plug burden is superior to blood eosinophils or FeNO; rather, these measures likely convey partially non-overlapping information. In practice, the most informative approach may be an integrated framework combining imaging (mucus plugs and airway morphometry) with inflammatory biomarkers (eosinophils, FeNO) and clinical features (exacerbation history and OCS dependence). We also recognize that the main limitations of our study are similar to those commonly seen in other real-life, single-centre investigations. These include the relatively small sample size, and the lack of randomization and placebo control. Nonetheless, the robustness of our findings lies in the approach of phenotypic stratification-guided therapy, supported by advanced imaging techniques.

Within this context, implementing standardized radiological criteria such as MPS could not only help to prevent disease progression and exacerbations, but also to significantly improve patients’ quality of life. These strategies align completely with the ethos of precision respiratory medicine, which seeks not only to control symptoms, but also to positively modify the natural course of severe asthma.

## Data Availability

The raw data supporting the conclusions of this article will be made available by the authors, without undue reservation.
